# Inferring bacterial cell size dynamics across media conditions

**DOI:** 10.1038/s41598-026-38811-1

**Published:** 2026-02-19

**Authors:** César Nieto, Claudia Igler, Abhyudai Singh

**Affiliations:** 1 Department of Electrical and Computer Engineering, University of Delaware, Newark, 19716 USA; 2 Institute of Integrative Biology, Zurich, Switzerland; 3 Division of Evolution, Infection and Genomics, School of Biological Sciences, University of Manchester, Manchester, M13 9PT UK

**Keywords:** Biological techniques, Biophysics, Cell biology, Microbiology

## Abstract

Under stable growth conditions, bacteria maintain cell size homeostasis through coordinated elongation and division. Changes in nutrient availability perturb these mechanisms, resulting in dynamic regulation of the target cell size. Using microscopy imaging and mathematical modeling, we studied how bacterial cell volume changes over the population growth curve and found that *Escherichia coli* and *Salmonella enterica*, in stationary phase, exhibit similar cell volume distributions irrespective of growth media. Resuspending cells in rich media resulted in a transient increase in cell volume to a media-dependent maximum cell volume after $\approx$ 2h before decreasing to the stationary phase cell size. Interestingly, stabilizing the growth phase through continuous fresh media supply sustained the size distribution. In poor media conditions, cell volume changed minimally over the growth curve, but cell width was markedly decreased. This cell volume dynamics along the growth curve can be related to a similar increase and decrease dynamics of the ratio between cell density ($\mathrm{OD}_{600}$) and cell numbers (CFU). We developed a simple mathematical modeling framework that predicted a time-varying division rate needed to capture the dynamics of the mean cell size across media conditions. The proposed analysis can be used for comparison of cell size regulation mechanisms across dynamic environments when single-cell tracking is not possible.

## Introduction

Bacteria regulate their cell size by modulating cell growth and division timing, often in response to environmental conditions. Recent studies suggest that cell size regulation is optimized to improve cell functionality ^1–5^. The efficiency of nutrient uptake and waste excretion, for example, is determined by the physical dimensions of a cell, including volume, shape, and surface-to-volume ratio^6^. Furthermore, fluctuations in cell size are an important source of noise in gene expression, potentially decreasing cellular functionality and fitness^7–9^. The physiological efficiency, in turn, affects the cell elongation and replication rates, i.e., the fitness of a population^10,11^. Therefore, controlling cell size is a key process for increase bacterial fitness, indicating evolutionary pressure to optimize its regulation across nutrient conditions ^12^.

To achieve this optimization, cells must deal with the constraints on chemical fluxes imposed by a particular growth environment ^2,13–15^.  In time-varying nutrient environments, cell volume appears to be determined by the balance between investment in cell-biomass production and cell division regulation ^16,17^. Hence, understanding the regulation of cell size under variable nutrient conditions is of particular relevance, since natural growth conditions are rarely constant^18^. In these variable environments, investigating the dynamics and mechanisms of cell size regulation is important to understand the synchronization of processes connected to growth and division, such as cell elongation, chromosome replication, and protein synthesis^16,17^, which are responsible for the rapid adaptation of bacterial cells to new nutrient environments^17,18^.

Population growth curves, or simply growth curves, are well-known examples of dynamical environments, where nutrient-depletion due to population growth leads to a slowing and eventually halting of cell growth, a stage known as stationary phase. Upon resuspension of cells in fresh nutrient media, the population then resumes exponential growth after a lag phase. Following cell size dynamics along the growth curve has recently been enabled by new developments in cell trapping, tracking and imaging. Such measurements have revealed significant changes in the volume of bacterial cells during different stages of the growth curve^16,19,20^, with larger cells found during exponential growth and smaller cells during nutrient-poor phases^21^.

In this study, using single cell microscopy and recent cell segmentation techniques on microscopy images^22^, we quantified the cell volume dynamics of two gram-negative rod-shaped bacterial species—*Escherichia coli* and *Salmonella enterica*—along the population growth curve for different nutrient media. We expand the scope of previous investigations by analyzing cell volume distributions over a wider set of changing and sustained media conditions, revealing complex trends in volume dynamics that can vary between nutrient media even when growth rate dynamics are similar.

We found that, regardless of the composition of the media, bacterial cells consistently exhibit a similar size in stationary phase, supporting the hypothesis of minimum bacterial volume ^23,24^. Upon resuspension in nutrient-rich media, cell volume dramatically increases, peaking during the early exponential phase. As observed previously ^16,20^, after reaching this peak, cell size gradually decreases during the transition into stationary phase. Under poorer growth conditions, we still observed a peak in cell size, but its magnitude was dependent on the specific nutrient composition, particularly the concentration of amino acid supplements. The timing of this peak in cell volume closely corresponded to the onset of a decrease in growth rate or, equivalently, the beginning of nutrient depletion. Interestingly, if the nutrient environment is continuously maintained through successive dilutions with fresh media, the entire cell volume distribution can be sustained for several hours. To quantify the coordination between growth and division that underlies these observed cell volume dynamics, we propose a simple, heuristic model ^25,26^ that uses population-level sizes and growth rate measurements without the need for single-cell tracking experiments. This approach allows us to understand and predict cell volume changes across diverse media conditions using readily available population metrics.

**Fig. 1:**
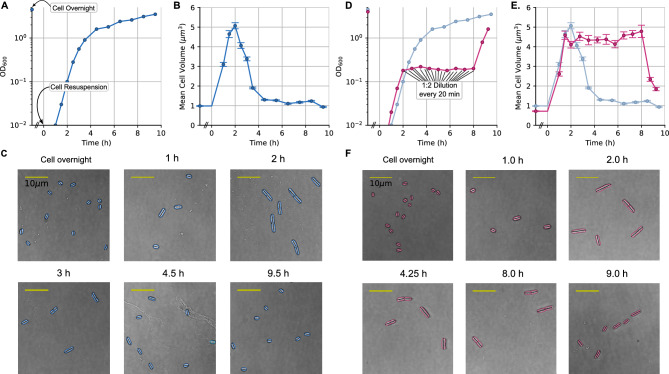
Cell volume dynamics of *E. coli* cells along the growth curve and for sustained exponential growth in rich media. (**A**) Optical density ($\mathrm{OD}_{600}$) and (**B**) mean cell volume of an *E. coli* culture along the growth curve (blue). (**C**) Examples of cells at different sampling points along the growth curve. (**D**) $\mathrm{OD}_{600}$ and (**E**) mean cell volume of an *E. coli* culture in sustained exponential growth conditions (pink), which was achieved through consecutive dilutions. The comparison with the regular growth curve is shown in light blue. (**F**) Examples of cells at different sampling points for sustained exponential growth. Colored contours show the cell segmentation and black lines show the dimension used as cell length.

## Results

### Cell volume peaks early in the growth curve

First, we studied *E. coli* MG1655 growth dynamics in Lysogeny broth (LB), a standard, yet undefined (i.e., exact composition is not replicable), rich medium. Cultures were grown in bulk under shaking conditions at 37$^\circ$C, with samples systematically collected for microscopy and optical density measurements (Methods). The initial sample was taken from an overnight culture which had depleted most nutrients and reached stationary phase. This cell culture was subsequently diluted 1:1000 ($t=0$) into fresh LB for regular sampling (Fig. 1A–C). Cell volume was estimated from segmented bacterial contours in microscopy images, approximating bacterial cells to sphero-cylinders (a cylinder capped by two hemispheres). Optical density of the population ($\mathrm{OD}_{600}$) was recorded at each sampling point (see Methods) to characterize the growth curve dynamics (Fig. 1A) and relate them to the estimated dynamics of the mean cell volume (Fig. 1B).

Bacteria from overnight cultures (stationary phase) had a small cell volume of $\approx 1\mu m^3$, which increased rapidly to $\approx 5\mu m^3$ over 2h after resuspension ($t=0$) (Fig. 1B,C). This peak volume was maintained only for $\approx$ 30min and then decreased over the next 2h until it stabilized at a volume slightly larger than that of overnight cells. As previously observed^16^, this peak in cell volume results in an opposing, albeit less pronounced, dip in the surface-to-volume ratio (Fig. S1). Analyzing the contributions of cell lengths and widths to volume changes, we observed an $\approx$ 1.5-fold increase in cell width and an $\approx$ 2.5-fold increase in cell length. The width increased $\approx$ 30min earlier than the length, while both decreased over similar time scales (Figs. S1A, S3). Cell length also showed a distinct broadening of the distributions and an increase in length variability between 1 and 3h after $t=0$ (Figs. S2A, S3), coinciding with the time frame of cell volume peak dynamics (Fig. 1B). Notably, the culture $\mathrm{OD}_{600}$ kept growing for several hours even after a decrease in cell volume was observed. However, during the entrance to stationary phase, the $\mathrm{OD}_{600}$ growth rate was not as high as the one observed in exponential phase, possibly due to the sequential depletion of nutrient sources^27^. We hypothesized that the observed cell size dynamics can be explained in two ways: either as a transient reaction to cell resuspension and awakening, or as a consequence of changes in nutrient levels.

### Sustained exponential growth preserves cell volume distributions

To test whether the decrease in cell volume was a transient event after leaving the stationary phase or rather dependent on the dynamic growth environment, we kept the cell culture under sustained exponential growth conditions for 6h. To do so, we diluted the culture 1:2 into fresh medium every 20min, beginning 2h after $t=0$, i.e., around the time when cell volume peaked (Fig. 1D). Under sustained exponential growth, cells maintained the peak mean volume of $\approx 5 \upmu \textrm{m}^3$ (Fig. 1E) and a broadened volume distribution (Fig. S4) for the entire 6h. When we stopped diluting the culture, the cell volume immediately began to decrease. Cell size homeostasis under steady conditions has previously been reported, mainly from microfluidic experiments^20,28^ and theoretically predicted^29,30^. Our experiments demonstrate that the transient peak in cell volume in nutrient-limited growth is a physiological adaptation to changes in the growth environment—most likely nutrient depletion—and not a transient effect of growth initiation. This is supported by the fact that cell volume distributions remain broadened as long as the nutrient environment is maintained (Fig. S4).

**Fig. 2:**
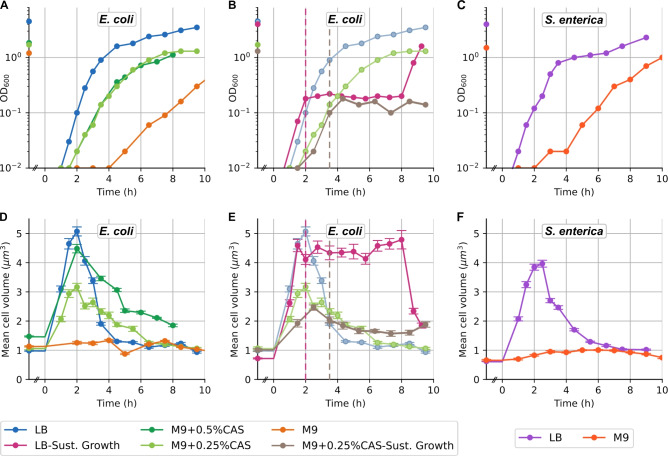
Cell volume dynamics across different media conditions for *E. coli* and *S. enterica*. (**A–C**) $\mathrm{OD}_{600}$ of cell culture and (**D–F**) mean cell volume over time in different conditions: (**A,D**) rich to poor media growth curves (blue, dark and light green to orange) and (**B,E**) sustained exponential growth in LB (pink) and M9 with 0.25% casamino acids (brown) for *E. coli* (regular growth curves are shown for comparison in light blue and light green, respectively); (**C,F**) rich (purple) and poor (red) media growth curves for *S. enterica*. Vertical dashed lines represent the time when the consecutive dilutions started in LB (Pink) and in M9 0.25%CAS (brown) respectively.

### Poor media conditions lead to almost constant cell volume

To understand how the dynamics of cell volume correlate with different nutrient media, we next investigated cell volume along the growth curve under poor media conditions. We used a defined, minimal medium (M9) with glucose as the sole carbon source^31^, which leads to slow cell growth, as cells need to synthesize amino acids rather than import them from the environment. Under these conditions, cell volume remained roughly constant throughout the growth curve around its stationary phase value of approximately $1\upmu \textrm{m}^3$ (Fig. 2A, D). Mean cell length increased slightly during growth in M9 glucose while cell width slightly decreased, overall keeping the cell volume (as well as surface-to-volume ratio) approximately constant (Fig. S1B).

As cell volume dynamics in rich and poor nutrient media differed drastically, we repeated these experiments in another gram-negative microorganism, *Salmonella enterica* LT2. As *S. enterica* is also rod-shaped and divides by symmetric binary fission, we would expect similar cell volume regulation as in *E. coli*. Indeed, we found peak dynamics in rich (LB) media and almost constant cell volume in minimal (M9 glucose) media (Figs. 2C, F, S1A) as seen with *E. coli* (Figs. 2A, D, S1A). 

### Cell volume peak height changes for different media compositions

The contrasting cell volume dynamics observed between rich and poor media conditions led us to research how cell size dynamics vary between different nutrient environments. We examined *E. coli* in two intermediate nutrient conditions: defined M9 medium with glucose, supplemented with either 0.25% or 0.5% casamino acids (partially digested amino acids). Surprisingly, both media produced similar $\mathrm{OD}_{600}$ growth curves but distinct cell volumes (Fig. 2A, D). The timing of the cell volume peak was consistent in both supplemented M9 media (around 2h after resuspension) while the height of the mean peak volume was significantly larger with 0.5% casamino acid supplementation compared to 0.25%. Specifically, cells grown in M9 with 0.5% casamino acids exhibited a peak cell volume similar to that observed in LB, whereas 0.25% casamino acid supplementation resulted in a peak volume approximately midway between poor and rich media ($\approx 3\mu m^3$) (Fig. 2D).

Similarly, our calculation of the surface-to-volume ratio revealed a considerably smaller change for M9 supplemented with 0.25% casamino acids, although this difference was less pronounced than for cell volume (Fig. S1B). Generally, for LB and M9 supplemented with casamino acids, the surface-to-volume ratio decreased during the initial 2h of growth and subsequently increased during the transition to stationary phase. This trend was opposite to cell volume changes, but occurred more slowly and with a lower magnitude of change (Fig. S1). In contrast, the surface-to-volume ratio in M9 glucose remained relatively constant throughout the growth curve and was consistently higher than in all richer media. This observation is related with the consistently smaller cell width in M9 glucose compared to the other media (Fig. S1).

In general, we identified a strong media dependence for both the maximum cell volume and the cell width, while the timing of the volume peak remained consistent under the conditions. This finding is further supported by a sustained growth experiment in minimal media supplemented with 0.25% casamino acids. In this experiment, the culture was diluted 1:2 in fresh medium approximately every 60 minutes, beginning 3.5 h after $t=0$ (that is, just after the cell volume began to decline) (Fig. 2B, E). Similarly to the sustained growth experiment at maximum volume in LB, we observed that the cell volume distribution stabilized for the duration of continuous dilution (3.5–9.5 h) and did not revert to the initial volume (Fig. 2B, E).

**Fig. 3:**
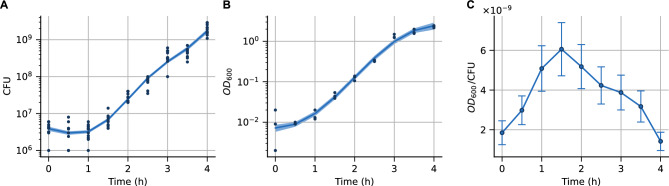
The ratio $\text {OD}_{600}$/CFU shows a similar peak pattern as cell volume over the growth curve. (**A**) Cell numbers as colony-forming units (CFU) (three biological replicates with four technical replicates each) and (**B**) $\mathrm{OD}_{600}$ as cells optical density along the growth curve in LB (three biological replicates), where lines show the best-fit curve to measurements. (**C**) The ratio between $\mathrm{OD}_{600}$ and cell numbers (CFU) over the growth curve. Confidence intervals were estimated using inference methods based on Gaussian processes^32^.

### The ratio between cell density ($\mathrm{OD}_{600}$) and cell numbers (CFU) shows similar peak dynamics as cell volume

The observed cell size dynamics along the growth curve suggests that during the early exponential phase, the increase in population $\mathrm{OD}_{600}$—which we use as a proxy for cell density - is largely driven by the increase in cell size rather than cell division. This would mean that relatively few new cells are generated while individual cells increase in size ^33,34^. This relationship then reverses in late exponential phase (Fig. 1A). Hence, we might expect that the relationship between $\mathrm{OD}_{600}$ measurements and the number of cells in the culture (colony forming units, CFU) is non-linear along the growth curve.

To test this hypothesis, we determined colony forming units (CFUs) as well as optical density ($\mathrm{OD}_{600}$) of *E. coli* cultures for 4 h after resuspension of overnight cultures in LB (Fig. 3A,B). We took measurements every 30 min, using three biological replicates plus four technical replicates each for CFU counts and three biological replicates each for $\mathrm{OD}_{600}$ (see Methods). Using inference methods based on Gaussian processes ^32^, we then determined the growth trends in $\mathrm{OD}_{600}$ (Fig. 3A) and CFU (Fig. 3B), and estimated the most probable ratio $\mathrm{OD}_{600}$/CFU (Fig. 3C). From this ratio, we found a similar peak pattern as for cell volume, although the peak occurs slightly earlier, at 1.5h instead of 2h after resuspension (Fig. 3C). Although the average peak height increases $\approx$ 3 times rather than the $\approx$ 5 times from Fig. 1A, the error range includes this 5-fold increase. Therefore, the $\mathrm{OD}_{600}$/CFU pattern follows a similar peak dynamics as the cell size changes.

The $\mathrm{OD}_{600}$/CFU peak indicates a discrepancy between the growth in optical density and cell number: Although cell elongation began soon after resuspension as visible in the increase in $\mathrm{OD}_{600}$ after 0.5-1h, cells were not yet dividing, meaning that CFU counts only increased after 1.5-2h. Hence, $\mathrm{OD}_{600}$ increased soon after inoculation, but population growth was delayed by $\approx$ 1h under these conditions. Conversely, after 2.5h, CFU numbers still indicate exponential population growth, while $\mathrm{OD}_{600}$ growth is already leveling off, indicating a decrease in $\mathrm{OD}_{600}$/CFU, and similarly, a decrease in cell volume (Fig. 3). In conclusion, our findings in cell size regulation have the following implications: the early increase in cell size suggests that growth is dominated by individual cell growth rather than proliferation (population growth), whereas later decreases in cell size when entering stationary phase imply ongoing cell proliferation despite a reduced overall growth rate.

**Fig. 4:**
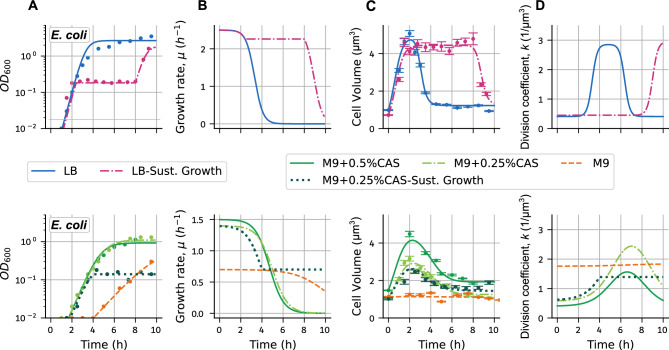
A model of cell volume dynamics shows the modulation of cell growth and division along the growth curve. (**A**) Population growth ($\mathrm{OD}_{600}$) over time for various media conditions as shown in different colors; points show measurements (Fig. 2A), solid lines are the fit to a logistic curve (with dilution when corresponds). (**B**) Growth rates obtained from fitted curves. During continuous dilutions, we approximate the growth rate to maintain its value just before these dilutions (**C**) Comparison of the mean cell volumes shown in Fig. 2D (points and error bars) and the prediction of the mean-field model of cell volume regulation (solid lines). (**D**) Dynamics of the division rate *k* over the growth curve in different media conditions. Rich media conditions are shown in the upper row, poorer media conditions in the lower row.

### A heuristic model relates changes in cell size to dynamic regulation of division

The dynamic relationship between cell density (growth) and cell numbers (division) implies that cell volume is regulated along the growth curve and in varying media conditions. To understand these changes, we used a differential equation model of cell size. Here, we refer to cell volume as cell size since our model does not explicitly take cell dimensions into account. We describe the change in cell size *s*(*t*) assuming that cells grow exponentially at a time-dependent rate $\mu (t)$:

1 $$\begin{aligned} \frac{ds(t)}{dt}=\mu (t) s(t). \end{aligned}$$

We can estimate $\mu (t)$ from the $\mathrm{OD}_{600}$ growth in our experiments (Fig. 4A,B). For simplicity, we ignore the phase of gradual decrease in $\mathrm{OD}_{600}$ growth rate during the transition to stationary phase (which might be caused by diauxic shifts) and focus on the main trends along the exponential phase in which most of the cell size dynamics occur ^35^. Therefore, we fitted a logistic curve to the $\mathrm{OD}_{600}$ measurements and used it to calculate the growth rate over time (Methods). As expected, $\mu (t)$ is high during early exponential phase and then decreases over the growth curve until it approaches zero with entry into the stationary phase. The growth curve fitting suggests that $\mu (t)$ is well described by a constant growth rate until $\approx$ 2h after cell resuspension in rich media conditions, which is the time point after which volume typically started to decrease as well (Fig. 4B).

We model cell size regulation by considering cell division as a discrete event, characterized by the halving of cell size at a rate determined by the division mechanism. Our model assumes this division rate is proportional to cell size, resulting in *adder* size control^36,37^. Cells following the *adder* strategy divide, on average, after adding a constant amount of size between birth and division, a mechanism widely associated with *E. coli*^38–40^ and other bacterial species^41–44^. The division rate also is considered to scale with cell size growth rate, following the form $k(t) \mu (t) s(t)$^25,26^, where *k*(*t*) is defined as the time-dependent division coefficient^25^. The division event can then be described as a discrete jump:

2 $$\begin{aligned} s \xrightarrow {k(t) \mu (t) s(t)} s/2. \end{aligned}$$

Using the mean-field approach (“Methods”) in which stochastic fluctuations are neglected, we can now describe the dynamics of the mean cell size $\langle s(t)\rangle$ ($\langle .\rangle$ indicating the mean at any given time point) through growth and division:

3 $$\begin{aligned} \frac{d\langle s(t)\rangle }{dt}=\underbrace{\mu (t) \langle s(t)\rangle }_{\text {cell growth}}-\underbrace{k(t)\mu (t)\frac{\langle s(t)\rangle ^ 2}{2}}_{\text {cell division}}. \end{aligned}$$

For a constant *k*, cell division and growth are synchronized and the mean cell volume will approach the steady-state value: $\langle s \rangle =\frac{2}{k}$. In this case, the mean cell volume depends only on *k* and is independent of the growth rate as the effect of $\mu$ on growth and division balances out.

The expression (3) suggest that we can infer *k*(*t*) knowing the (mean) cell size, $\langle s\rangle$, and growth rate, $\mu$ (estimated from $\mathrm{OD}_{600}$). This inference (Methods) reveals that *k* is not constant over the growth curve under most media conditions (Fig. 4D): *k* starts low, then increases when the growth rate begins to decrease, and plateaus as the growth rate is reaching its minimum value. We chose an approximation of *k* that decreases again over the stationary phase, ’resetting’ to its basal value once cells stop growing, i.e. *k* is the same at $t=0$ and $t\rightarrow \infty$. The steepness of the increase in *k* differs between media conditions, highlighting that division is determined by a combination of growth rate and target cell volume, where the former can be similar between different growth conditions while the latter is usually different. For example, cells in M9 supplemented with 0.25% and 0.5% casamino acids have similar growth rates, but the division parameter *k* is higher for 0.25% supplementation, which results in smaller cells (Fig. 4B-D). Interestingly, for cells in M9 without supplements the division parameter remains at the relatively high steady state value of $k=2/\langle s\rangle$. This suggests that the degrees of freedom for the division parameter are limited in slow growth conditions, e.g. by limited nutrient intake. Finally, in sustained growth experiments, $\mu$ and *k* are constant and cell size changes are minimal (Fig. 4B–D). The observed slight deviation from constant cell size in sustained growth experiments is likely related to difficulties with exact dilution timing and resulting deviations in cells optical density (Fig. 4C).

## Discussion

In this study, we investigated cell volume dynamics in two rod-shaped bacteria, *E. coli* and *S. enterica*, revealing a strong dependence of cell size regulation on media conditions. We explored the regulation of bacterial cell size along the growth curve across a range of nutrient environments, from very poor (M9 minimal medium) to rich (LB), and propose a simple method to analyze the behavior of growth and division mechanisms that regulate cell size under fluctuating environmental conditions. These tools have particular relevance when single-cell tracking is not available. Cell size estimation can be extended beyond cell microscopy to experiments involving techniques such as flow cytometry^45,46^, coulter counter^47^, and microfluidic resonators^48^. Our approach also has the advantage that it does not require measurements or knowledge of the specific mechanisms that affect cell division dynamics. Determining the division parameter *k* through our approach can be a simple and meaningful way of testing hypotheses on, for example, the effect of environmental changes^17^ or mutations on cell size regulation^49^.

Here, we investigated the remarkable plasticity and adaptability of bacterial cells in regulating their size in response to environmental changes by balancing resource allocation between cell growth and division in response to nutrient availability. One of the primary characteristics typically associated with changes in nutrient availability is the growth rate of bacterial cells, which is closely linked with cell size^21,50^. We show that the division rate depends on media conditions in a different manner than the growth rate as we found conditions with similar growth rate dynamics (M9 with 0.25% and 0.5% CAS), but different cell volumes. Generally, we observed that in all nutrient-rich environments (LB broth or M9 media with casamino acids), cell volume consistently reached its peak about 2h after resuspension into fresh media, regardless of how fast they were growing at their maximum rate. However, the size of the cell volume peak directly depended on how rich the medium was, with richer media leading to larger maximum cell volumes. This was especially clear when comparing different concentrations of amino acid supplements in M9 media. After comparing cell volume and growth rate changes over the growth curve, we conclude that the average cell volume started to decrease as soon as the population growth rate started to slow down. This strongly suggests that a decrease in growth rate, likely due to nutrients becoming scarce, triggers the decrease in cell volume until it eventually returns to a common stationary phase volume ($\approx 1\mu m^3$).

Cell volume is just one of the dimensions of cell size dynamics that we considered. Other measures are cell length and width, which were determined from the segmentation masks of the microscopy images ^10^. In agreement with previous research ^16,51^, we found that cell width increased earlier than cell length, with a delay of $\approx$ 30min (Fig. S1). Surprisingly, there seemed to be more cell width variation between media conditions, although changes over the growth curve were greater in cell length (Fig. S1). For example, the difference in cell volume dynamics between cells grown in M9 supplemented with 0.25% and 0.5% casamino acids, was mainly caused by cells being significantly wider in the latter, while lengths were similar (Fig. S1). Such changes are particularly interesting, as bacteria can use changes in cell shape, specifically changes in surface-to-volume ratio, as a means of antibiotic resistance by reducing the intracellular or membrane concentration of an antibiotic ^52^. Previous work has also used some of these other cell size measurements to model regulation of cell division, e.g. focusing on cell length, cell surface area or the surface area-to-volume ratio ^10,16,28,53^.

Poor medium (M9 without casamino acids) presented a special case, in which cells did not show appreciable changes in cell volume or surface-to-volume ratio over the growth curve (Fig. S1B,C). This was mainly due to the constant cell width throughout the growth curve, although the cell length increased slightly (Fig. S1B,C). Interestingly, cells were thinner and longer than cells with similar volume in other growth conditions (this volume is usually reached during the transition to stationary phase) (Fig. S1B,C). In *S. enterica*, the increase in cell length in M9 minimal media was more pronounced than in *E. coli*, giving an overall slight increase in cell volume, which only decreased again $\approx$ 7h into the experiment (Fig. S1) at which point the $OD_{600}$ growth rate also started to decrease (Fig. 4B). These results suggest that the conserved 2h peak in all other media conditions was coincidental and support that the peak is related to the change in growth rate, potentially caused by nutrient depletion. However, given the small change, lack of a proper peak in volume dynamics and very slow growth rate, the poor medium does not allow for conclusive evidence about the regulation of cell volume dynamics. Furthermore, our model showed that the division parameter did not change over most of the growth curve in M9 with glucose (Fig. 4E), suggesting little change in the mechanisms of size regulation.

During the exponential growth phase, we observed a high growth rate but a relatively low division (proliferation) rate. This suggests that cell volume drastically changes during the first two hours of the growth curve are due to cell growth rather than just cell division. In fact, our results (Fig. 3) show that the relationship between cell number (CFU) and cells optical density ($\text {OD}_{600}$) was not constant. This indicates that for the first $\approx 1.5$ hours, the increase in $\text {OD}_{600}$ was primarily due to an increase in individual cell volume, not an increase in cell count. We estimate that the maximum $\text {OD}_{600}$/CFU was achieved after approximately 1.5h. However, the peak in cell size occurred roughly half an hour later, corresponding to 3-4 cell doublings (Fig. 1A, 3C). The magnitude of the observed changes in $\text {OD}_{600}$/CFU ratio largely agree with those of cell volume changes if measurement errors are taken into account. While we avoided the saturation in optical density by diluting the sample before measuremnent (see Methods), additional variables that we are not accounting for, such as the change in biomass density can modify the interpretation of $\text {OD}_{600}$ along the growth curve^54^. Further, $\text {OD}_{600}$ does not directly reflect cell dry-mass density^55^, meaning that our results primarily show a qualitative agreement between cell size regulation and the ration optical density over cell population changes along the growth curve. Nonetheless, the discrepancy between optical density and actual cell count has significant practical implications for microbiology experiments. Relying solely on optical density can lead to a substantial overestimation of cell numbers, especially during dynamic growth phases. This non-linearity also means that the accuracy of cell number estimates using $\text {OD}_{600}$ heavily depends on consistent experimental conditions and precise measurement timing to ensure comparability ^33,54,56^.

Most of our analysis focused on the mean cell size behavior, but we also observed interesting patterns in the noise or variability in cell size regulation between individual cells (Fig. S2). We observed that the awakening of cells after resuspension led to a broadening of the cell volume distribution that lasted throughout most of the peak dynamics (Fig. S3). This variability appeared to come from variability in cell length rather than cell width (Fig. S2, S3), indicating heterogeneity in the timing of the first division between cells. The increase in cell volume and length noise did not seem to be a transient phenomenon, as sustained growth conditions preserved not only the cell volume but also the broadness of the distribution at different points along the growth curve (Figs. 2B, E, S4). In general, the increase and decrease in the mean and variability of cell volume appear to be related to a change in the nutrient environment, which could be caused by differences in adaptation speed of intracellular machinery to environmental conditions between the individuals in a population ^57^. The relationship between cell size and media conditions - and the resulting cell size heterogeneity—is highly relevant to understanding the behavior of natural microbial communities, but also clinical phenotypes, such as resistance to antibiotics and persister cells^20,52^.

Our study describes size regulation trends across different growth conditions for two rod-shaped bacterial strains. As the bacterial strains used here share certain similarities, this does not fully demonstrate the generality of our results, but provides a valuable foundation for the design of future experiments aimed at testing a broader range of conditions or strains, which can be done using the versatile analytical method developed here. Our findings suggest several new perspectives for further experiments and modelling: First, the convergence to a common cell size during stationary phase across diverse growth conditions remains unexplained by current growth-division allocation models^17^. This could suggest a potential minimal division threshold - below which cell division is disabled^24,58^—that might be linked to chromosome replication initiation. This hypothesis could be further studied by labeling replication origins and following cell size dynamics in fluctuating environments as shown in recent studies ^23^. Second, the higher variability in cell size at bigger volumes is intriguing and a deeper understanding of its origin could shed light on the constraints governing cell size regulation. Previous studies indicate that understanding stochasticity in cell division mechanisms allows us to distinguish between various model types that predict similar mean dynamics^58^. Coupling such modelling frameworks with time-dependent dynamics has proven useful for characterizing cell size homeostasis in diverse systems such as cancer cells^49^, yeast^53,59^ and different bacterial species^30^.

Modeling the growth curve itself presents inherent challenges. Although our study used a logistic model for population dynamics, alternative approaches such as the Gompertz model may offer more accurate fits in certain contexts (e.g., tumor growth)^60^. Gompertz models are particularly relevant for incorporating diverse cell phenotypes (e.g., quiescent or proliferating subpopulations, which vary in proportion along the growth curve^61^) or when accounting for subpopulations using different nutrient sources (as in diauxic growth^62^). In Fig. S5, we show that a Gompertz function fits the population growth better during late growth curve (Fig. S5A), but its poor fit to the exponential phase—where the majority of cell size dynamics occur—limits its utility. Hence, even though the Gompertz model predicts similar dynamics for the division parameter (Fig. S5D), it does not fit cell size as accurately as the logistic approximation (Fig. S5C) due to the inaccurate representation of the exponential phase.

Our work highlights how experiments using varying media conditions can reveal changes in division mechanisms that uncover hidden features^17^ and their influence on growth transition kinetics^13^. Future research could couple mechanistic models of nutrient consumption with experiments that systematically modulate nutrient type and concentration (e.g., nitrogen and carbon sources) to gain a more comprehensive picture of cell volume mean and noise dependence on nutrient composition^63^. However, such models can be more complex and difficult to parametrize. For this reason, we focused here on providing a simple model that allows an easy, conceptual comparison of cell size regulation across different environments and genotypes. 

## Materials and methods

### Bacterial growth conditions and media

Experiments were carried out using the * E. coli* K-12 substrain MG1655 and the *S. typhimurium* LT2 derivative, TH437. Bacterial cells were grown overnight at 37C with shaking and aeration in 15 ml culture tubes filled with 2 ml of growth medium. Minimal media consisted of 1X M9 salts, 1mM thiamine hydrochloride, 0.4% glucose, 2mM MgSO4, 0.1mM CaCl2, without or with 0.5% or 0.25% casamino acids. M9 salts, casamino acids and LB media were autoclaved, and other ingredients were sterilized with filter sterilization.  Overnight cultures were diluted 1:1000 v/v into 10ml of the same medium and grown on the shaking incubator with aeration at 37C. Samples were taken at 30- or 60-min intervals and imaged under the microscope. For cultures that were kept in an early exponential phase in rich media, half of the culture was diluted with the same amount of fresh media approximately every doubling time and sampled for imaging at every second dilution.

### Single-cell microscopy

Between 1 and 6 $\mu l$ of the sample (diluted 2- or 4-fold at later time points to avoid cell clusters) were spotted on an agar pad (made of minimal media with 1.5% agarose) and left to dry for a few minutes. The agar pad was inverted and placed into a microscopy dish ($\mu$-Dish 35mm, low; Ibidi). The microscope dish was mounted on an inverted microscope stage (Eclipse Ti2-E, Nikon) and bright-field images were taken with a Nikon DS-Qi2 camera using a 100$\times$ oil immersion objective (Plan Apo $\lambda$, N.A. 1.45, Nikon).

### Optical density measurements

At each sampling point, the optical density at $600\text { nm}$ ($\text {OD}_{600}$) was measured using a spectrophotometer with a $1\text { ml}$ sample volume. To ensure measurements fell within the linear range of the instrument and to minimize errors caused by culture crowding and light scattering effects, samples with an $\text {OD}_{600}$ greater than 0.4 were diluted. These samples were diluted with fresh growth medium until the measured $\text {OD}_{600}$ was below 0.4. The true culture $\text {OD}_{600}$ was subsequently determined by multiplying the measured value by the corresponding dilution factor.

### CFU count plating

For cell number counts, we diluted overnight cultures of * E. coli* K-12 substrain MG1655 1:1000 into fresh LB media. 3 biological replicates were measured every 30min for 4h to obtain $OD_{600}$ via a spectrophotometer (see above) and CFU by plating. For plating, we used the running droplet method where 10ul of each sample dilution ($10^0-10^{-7}$) are dropped onto a square plate, which is then tilted so that droplets can run down to half of the plate. We plated each dilution twice and used all dilutions with countable colony numbers for analysis. 

### Segmentation and analysis of microscopy data

We estimated the cell volume from the bright-field microscopy images by segmenting cells using the pixel-classification module of Ilastik (v.1.3.3)^22^. As each nutrient medium influenced the cell shape differently along the growth curve, we trained an Ilastik neural network for each condition. The training process involved outlining an arbitrary set of cells to define the contours and conducting manual checks to resolve instances where cell clusters posed segmentation challenges. We devoted special attention to exponential phase images, to differentiate between cells that had not yet undergone division and cells that had undergone division but remained physically connected. This is exemplified in Fig. 1C, F at the 2-h mark.

From the estimated contours we measured the projected area $A_p$, which corresponded to the number of pixels inside the contour times the pixel area ($\approx$ 0.005 $\upmu \textrm{m}^2$/pixel). We defined the cell length *L* as the longest side of the minimum-bounding rectangle of the contour. The projected area $A_p$ and the cell length *L* are related to the effective cell width *w* through the projected area of a capsule:

4 $$\begin{aligned} A_p= w(L-w)+\pi \left( \frac{w}{2}\right) ^2. \end{aligned}$$

Thus, from $A_p$ and *L*, *w* is estimated by solving Eq. (4). The cell surface area *A* and volume *V* can then be estimated from *L* and *w* as follows:

5 $$\begin{aligned} A&=\pi Lw;\nonumber \\ V&=\pi (L-w)\left( \frac{w}{2}\right) ^2+\frac{4}{3}\pi \left( \frac{w}{2}\right) ^3=\frac{\pi Lw^2}{4}-\frac{\pi w^3}{12}. \end{aligned}$$

After obtaining these dimensions for all segmented cells, we filtered the outliers (usually imaging artefacts) considering only cells satisfying the following criteria:

- Cell width greater than 0.35 $\mu$m.
- Cell length greater than 1.05 $\mu$m and less than 10 $\mu$m.
- Cell aspect ratio (*L*/*w*) greater than 1 and less than 7.
- Cell area greater than 0.73 $\mu m^2$.

Further, for each time point, we applied a final outlier filtering step. We discarded any cells where the deviation in the log-transformed cell volume from the log-transformed population mean cell volume exceeded three times the log-transformed population standard deviation of the cell volume. This assumes that the cell size distributions follow a log-normal distribution and therefore, this range should include approximately 99.7% of the data. Statistics and confidence intervals were estimated using Bayesian methods^64^.

### Estimation of OD and CFU time curves

To estimate the ratio OD/CFU over 4h after resuspension, we ran an inference algorithm^32^ separately on the replicates of cell number and population $\text {OD}_{600}$ respectively. As a result, we obtained the most probable trajectory of the mean with its 95% confidence interval (Fig. 3A, B). Given the most probable mean OD *x* with its confidence interval $\Delta x$, and the most probable CFU *y* with its confidence interval $\Delta y$, the most probable ratio *z* and its confidence interval $\Delta z$ were estimated using the formulas:

6 $$\begin{aligned} z= & \frac{x}{y}\nonumber \\ \Delta z\approx & (\Delta x)\Bigg |\frac{\partial z}{\partial x}\Bigg |+(\Delta y)\Bigg |\frac{\partial z}{\partial y}\Bigg |= \Bigg |\frac{\Delta x}{y}\Bigg |+\Bigg |\frac{x}{y}\frac{\Delta y}{y}\Bigg |, \end{aligned}$$

Standard methods for propagation of uncertainty were used for this estimation.

### Mean-field cell size dynamics

A more detailed approach to the theoretical model was published recently^36^. Briefly, assuming that cells grow following the dynamics in Eq. (1) and halve their size at each division, the expected value of any arbitrary function of cell size *f*(*s*) follows the dynamics:

7 $$\begin{aligned} \frac{d \langle f(s) \rangle }{dt}= \left\langle \mu s \frac{df(s)}{ds} + k\mu s\left( f(s/2)-f(s) \right) \right\rangle . \end{aligned}$$

To estimate the *n*-th moment of cell size, we replaced $f(s)=s^n$ and obtained the differential equation governing the dynamics of $\langle s^n\rangle$:

8 $$\begin{aligned} \frac{d \langle s^n \rangle }{dt}= \left\langle \mu s \frac{d(s^n)}{ds} + k\mu s\left( \left( \frac{s}{2}\right) ^n-s^n \right) \right\rangle = n\mu \langle s^n\rangle - k\mu \left( 1-\frac{1}{2^n}\right) \langle s^{n+1}\rangle . \end{aligned}$$

The main issue with equation (8) is that the dynamics of $\langle s^n\rangle$ depend on $\langle s^{n+1}\rangle$. This problem is known as unclosed moments dynamics^65^ and has been explored recently^66^. The mean-field approximation neglects the random fluctuations of *s* and its correlation with other variables. Therefore, we approximate the second-order moment as $\langle s^2\rangle \approx \langle s\rangle ^2$ and ignore the equations for higher-order moments to obtain the equation (3) in the main text.

### Fitting procedure for the division coefficient

To simplify our approach, we first fitted the OD curves to a logistic curve of time. This simplification assumes that the growth rate is proportional to the nutrients and that the rate of nutrient depletion is proportional to the cell density, which is simplified as proportional to the culture $\text {OD}_{600}$. In summary, we assumed that the biomass *B* follows:

9 $$\begin{aligned} \frac{dB}{dt}=\mu _{max}B\left( 1-\frac{B}{B_{max}}\right) , \end{aligned}$$

where $\mu _{max}$ and $B_{max}$ are the maximum growth rate and the carrying capacity of the growth medium, respectively. We adjusted the OD dynamics to the solution of (9):

10 $$\begin{aligned} B(t;t_s,B_{max},\mu _{max})=\frac{B_{max}}{1+e^{-\mu _{max}(t-t_s)}}, \end{aligned}$$

where the free parameter $t_s$, depending on the initial conditions of *B* can be interpreted as the time for entering stationary phase. The parameters $t_s$, $B_{max}$ and $\mu _{max}$ were fitted to data by minimizing the error function:

11 $$\begin{aligned} \sum _i(\ln (B_i)-\ln [B(t_i;t_s,B_{max},\mu _{max})])^2, \end{aligned}$$

in which $B(t_i;t_s,B_{max},\mu _{max})$ is calculated from the Eq. (10) at $t=t_i$ for each *i*th point. Note that the error function is the Euclidean distance between the logarithm of the data and the logarithm of the model with $t_s,B_{max},\mu _{max}$ as fitting parameters. After fitting, from the equation for *B* over time (10) we could then obtain the growth rate over time:

12 $$\begin{aligned} \mu (t)=\frac{1}{B}\frac{dB}{dt}=\frac{\mu _{max}}{1+e^{\mu _{max}(t-t_s)}}. \end{aligned}$$

The division dynamics were assumed for simplicity to be described by the division coefficient that follows the dynamics:

13 $$\begin{aligned} k(t)=k_0+(k_{max}-k_0)\left( \frac{1}{1+e^{-\lambda (t-t^*_1)}}-\frac{1}{1+e^{-\lambda (t-t^*_2)}}\right) , \end{aligned}$$

with four free parameters: the basal coefficient $k_0$, the maximum coefficient $k_{max}$, the rate of change $\lambda$ and the time of reallocation $t^*_1$ is the value of time where *k*(*t*) reaches the halfway point between $k_0$ and $k_{max}$ and $t^*_2$ is the time instant in which the division parameter decreases again. This assumption was considered in order to maintain the same division parameter at the beginning and the end of the experiment since both of them correspond to the same condition: stationary phase. Proposing a set of parameters $k_0$, $k_{max}$, $\lambda$, $t^*_1$ and $t^*_2$, we can numerically integrate the mean cell size $\langle s\rangle$ from the differential equation (3) with initial condition $\langle s\rangle |_{t=0}=s_1$. The mean cell size at time $t=0$ was taken as the mean cell size from overnight cultures. The optimized parameters for *k*(*t*) were selected after a comparison between the observed mean sizes $\{\langle s\rangle _1,\cdots \langle s\rangle _N\}$ and the predictions $\{\langle s(t_i)\rangle ,\cdots \langle s(t_i)\rangle _N\}$, where $\langle s(t)\rangle$ is the numerical solution of Eq. (3). This optimization minimized the error function:

14 $$\begin{aligned} \sum _i\left( \frac{\langle s\rangle _i-\langle s(t_i;k_0,k_{max},\lambda , t^*_1,t^*_2)\rangle }{\langle s\rangle _i}\right) ^2. \end{aligned}$$

Note that this error function corresponds to the square of the difference in mean cell size between theory and experiment divided by the observed mean cell size.

#### Modification for accounting the media dilutions

This theoretical approach is slightly modified in experiments with consecutive media dilution. In that context, the $\text {OD}_{600}$ derivative is set to zero during the time-span in which the dilutions are performed and the growth rate is kept constant in the value just before the dilutions started. 

## Supplementary Information

Supplementary Figures.

## Data Availability

The data set and data processing scripts are publicly available at https://zenodo.org/records/13208280^67^.
