# Supplementary material for: Inferring bacterial cell size dynamics across media conditions
Source: Sci Rep. 2026 Feb 19;16:9883. doi: 10.1038/s41598-026-38811-1 (PMC13018629; doi:10.1038/s41598-026-38811-1)
Supplement: Supplementary file 1 — Supplementary Figures. [file 41598_2026_38811_MOESM1_ESM.pdf]

Supplementary Material: Inferring bacterial cell size dynamics across media conditions

César Nieto[a,*], Claudia Igler[b,c,*], and Abhyudai Singh[a,†]

[a]Department of Electrical and Computer Engineering, University of Delaware, Newark, DE 19716, USA.

[b]Institute of Integrative Biology, ETH Zurich, Zurich, Switzerland

[c]Division of Evolution, Infection and Genomics, School of Biological Sciences, University of Manchester, Manchester M13 9PT, UK

[*]Authors contributed equally

[†]Correspondence: absingh@udel.edu

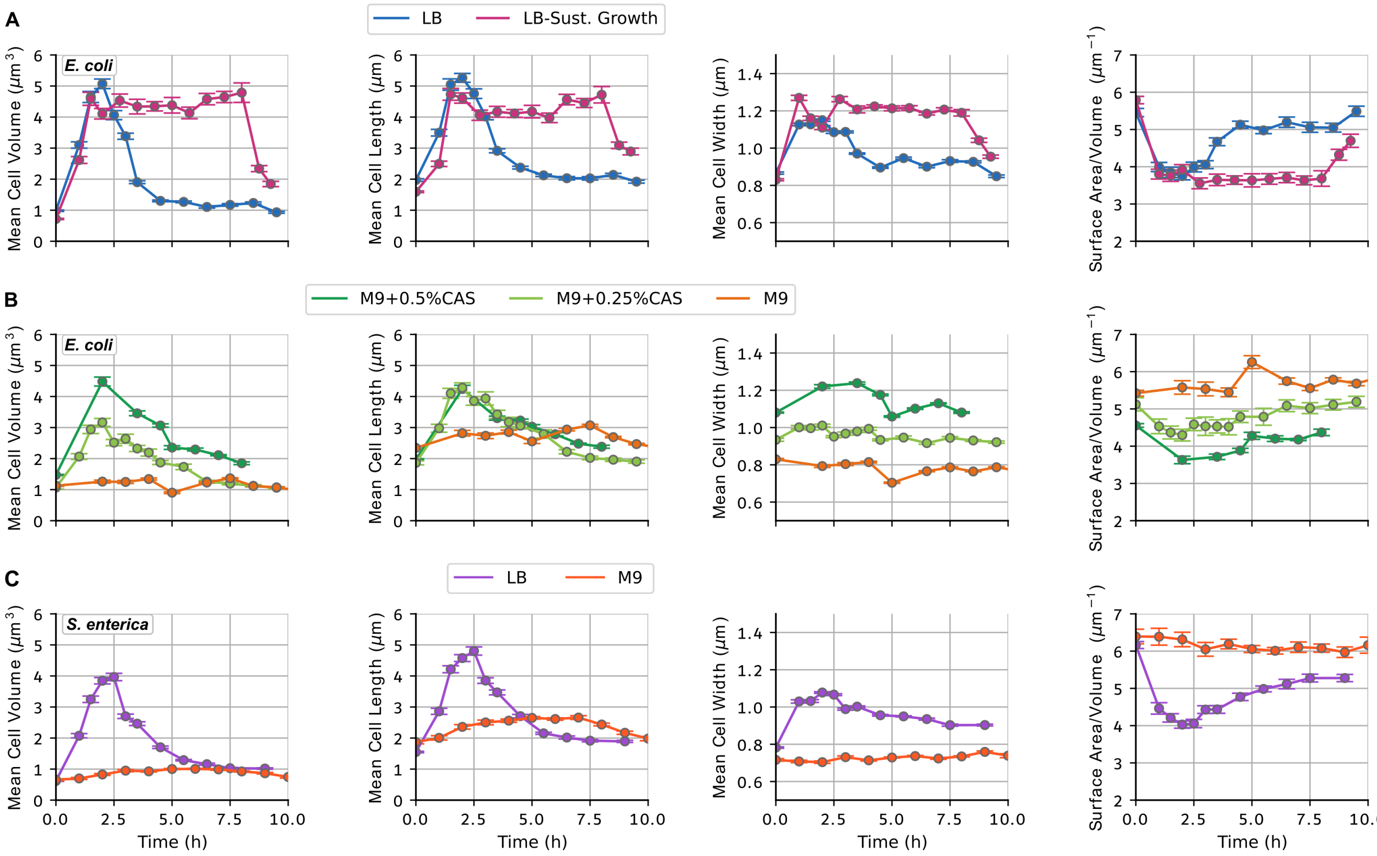

Figure S1: **Cell volume, length, width and surface-to-volume ratio dynamics across different media conditions for *E. coli* and *S. enterica*.** Mean cell volume, length, width, and surface-to-volume ratio of (**A**) *E. coli* cell cultures at different sampling points over time in LB (nutrient-limited in blue and sustained exponential in pink), (**B**) *E. coli* cell cultures at different sampling points over time in M9 glucose (orange) or M9 glycerol supplemented with 0.25% or 0.5% casamino acids (light and dark green), or (**C**) *S. enterica* cell cultures at different sampling points over time along rich (purple) and poor (red) media growth curves. Overall, we found that changes in cell length were more pronounced than changes in cell width. Cell width was consistently smaller in minimal media with glucose than in richer media. Interestingly, for minimal media with different supplements, cell width was more distinct than cell length (B). LB media showed the biggest change in cell length and led to the strongest drop in the surface area-to-volume ratio.

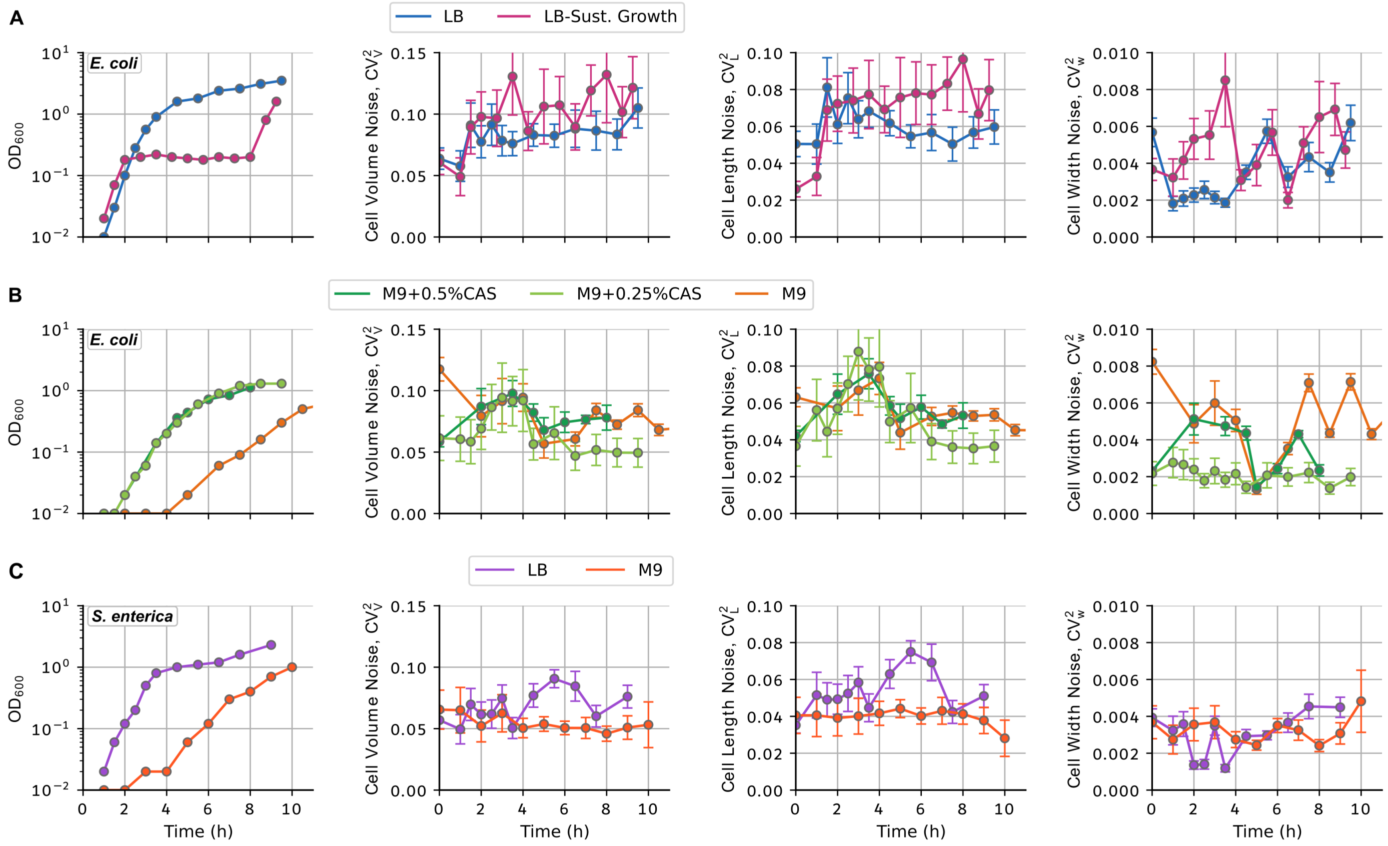

Figure S2: **Noise in cell volume, length and width across different media conditions for *E. coli* and *S. enterica*.** OD_{600} and noise (quantified by the coefficient of variation squared CV^2) of cell volume, length and width for (**A**) *E. coli* cell cultures at different sampling points over time in LB (nutrient-limited in blue and sustained exponential in pink), (**B**) *E. coli* cell cultures at different sampling points over time in M9 glucose (orange) or M9 glycerol supplemented with 0.25% or 0.5% casamino acids (light and dark green), or (**C**) *S. enterica* cell cultures at different sampling points over time along rich (purple) and poor (red) media growth curves. We found that the noise in cell length was larger than the noise in cell width.

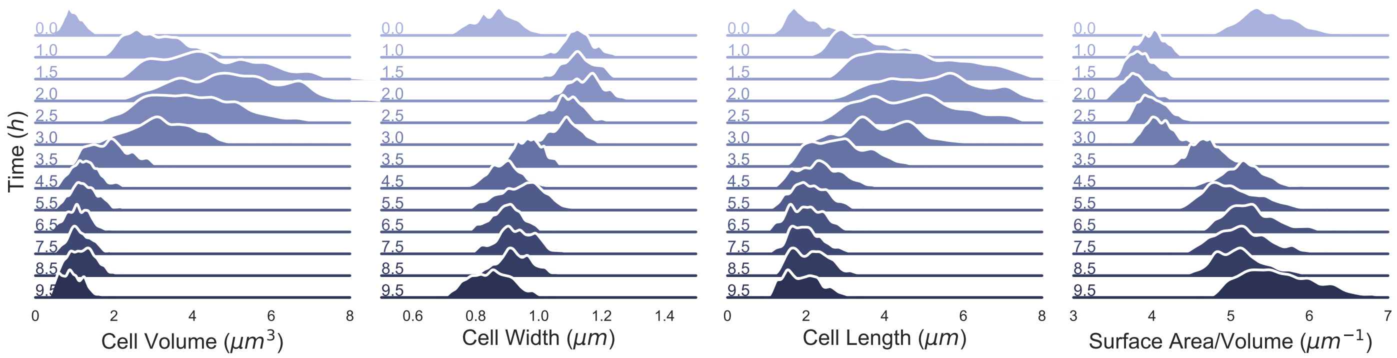

Figure S3: **Distributions of cell volume, width, length and surface-to-volume ratio for *E. coli* over the growth curve in LB.** Full distributions of cell volume, width, length and surface-to-volume ratio estimated from segmented cells in microscopy images are shown along a growth curve in LB. t = 0 at the top indicates the overnight sample and subsequent samples are shown on the vertical axis. We found a broadening of the cell volume and length distributions around the time of the peak (1.5-3h). Cell width distributions did not become broader but the average cell width shifted earlier than that of the cell length.

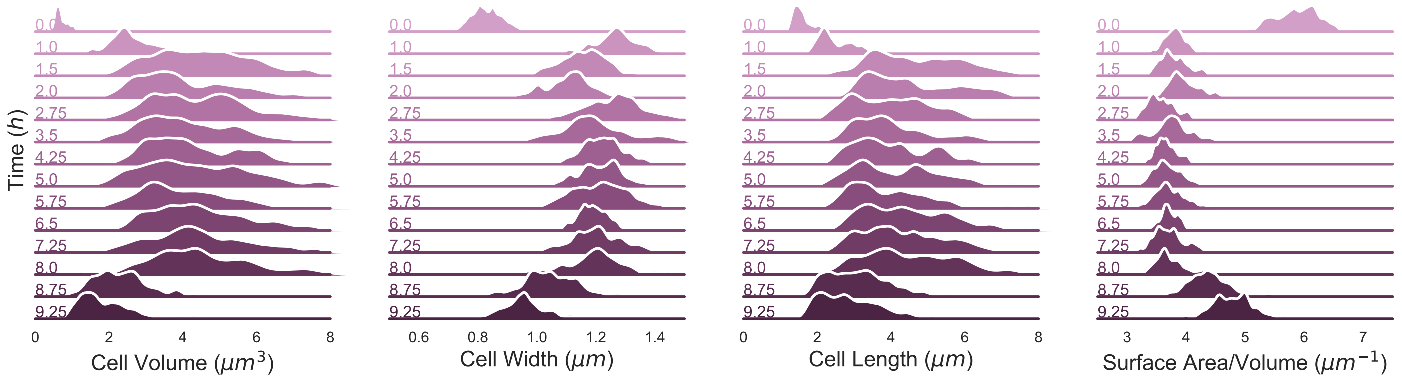

Figure S4: **Distributions of cell volume, width, length and surface-to-volume ratio for *E. coli* in sustained exponential growth in LB.** Histograms of cell volume, width, length and surface-to-volume ratio estimated from segmented cells in microscopy images are shown for sustained exponential growth in LB. t = 0 at the top indicates the overnight sample and subsequent samples are shown on the vertical axis. Sustaining exponential growth in LB media preserved the cell volume, length and width distributions. Surprisingly, the broadness of the cell volume and length distributions was also preserved, indicating that the noise in cell size was not caused by a transition from poor to rich medium.

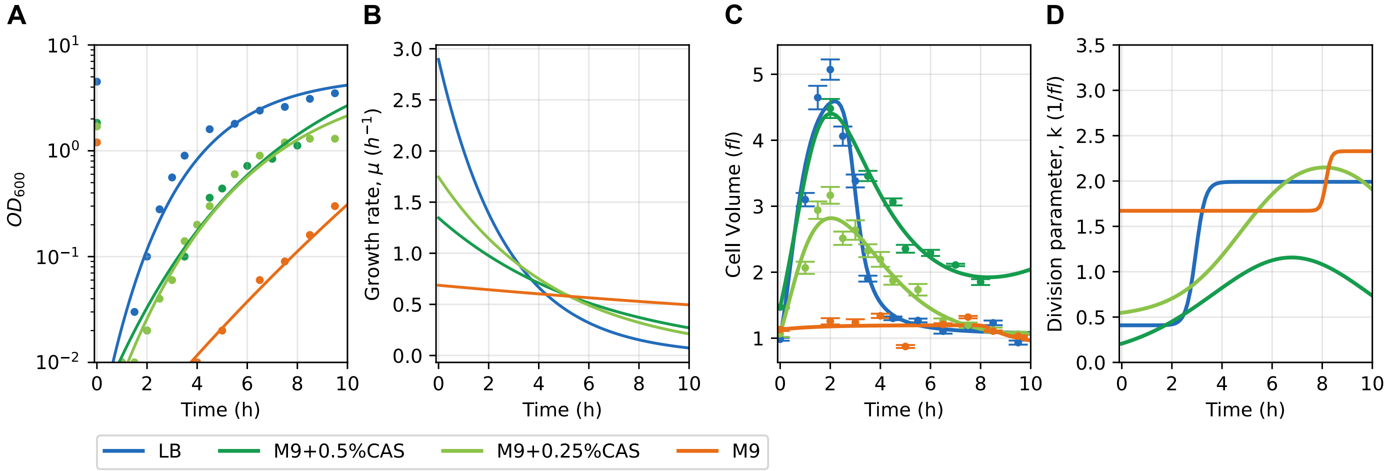

Figure S5: **Cell division dynamics with growth rate fitted to a Gompertz function**. (**A**) Cell density (OD_{600}) over time for various media conditions as shown in different colors; points show measurements, solid lines are the fit to a Gompertz curve. (**B**) Growth rates obtained from fitted curves. During continuous dilutions, we approximate the growth rate to maintain its value from just before these dilutions. (**C**) Comparison of the mean cell volumes in Fig. 2D (points and error bars) and the prediction of the mean-field model of cell volume regulation (solid lines). (**D**) Dynamics of the division rate k over the growth curve in different media conditions.
